# Mapping the burden of diabetes in five small countries in Europe and setting the agenda for health policy and strategic action

**DOI:** 10.1186/s12961-020-00665-y

**Published:** 2021-03-29

**Authors:** Sarah Cuschieri, Elena Pallari, Natasa Terzic, Ala’a Alkerwi, Árún Kristín Sigurðardóttir

**Affiliations:** 1 Department of Anatomy, Faculty of Medicine and Surgery, University of Malta, Msida, Malta; 2 MRC Clinical Trials and Methodology Unit, University College London, London, UK; 3 Center for Health System Development, Institute of Public Health of Montenegro, Podgorica, Montenegro; 4 Service épidémiologie et statistique, Direction de la Santé, Luxembourg, Luxembourg; 5 School of Health Science, University of Akureyri, Sólborg, Iceland; 6 Akureyri Hospital, Akureyri, Iceland

**Keywords:** Type 2 diabetes, Noncommunicable diseases, Healthcare systems, Health policy, Healthcare delivery, Global burden of diseases, Small countries

## Abstract

**Background:**

Diabetes is a global epidemic affecting every country. Small countries, however, face distinctive challenges related to their health system governance and their ability to implement effective health systems’ reforms. The aim of this research was to perform a comparative assessment of existing diabetes management practices at the population level and explore governmental-related policy for Cyprus, Iceland, Luxembourg, Malta and Montenegro. This is the first time that such an evidence-based review study has been performed in the field of diabetes. The overall purpose was to set the agenda for health policy and inform strategic actions for small countries that can benefit from dealing with the diabetes epidemic at a country level.

**Methods:**

We collected data and synthesized the evidence on dealing with diabetes for each of the five small European countries according to the (1) epidemiology of diabetes and other related metabolic abnormalities, (2) burden of diabetes status and (3) diabetes registers and national plans. We collected data by contacting Ministry representatives and other bodies in each state, and by searching through publicly available information from the respective Ministry of Health website on strategies and policies.

**Results:**

Diabetes rates were highest in Cyprus and Malta. National diabetes registers are present in Cyprus and Montenegro, while national diabetes plans and diabetes-specific strategies have been established in Cyprus, Malta and Montenegro. These three countries also offer a free holistic healthcare service to their diabetes population.

**Conclusions:**

Multistakeholder, national diabetes plans and public health strategies are important means to provide direction on diabetes management and health service provision at the population level. However, political support is not always present, as seen for Iceland. The absence of evidence-based strategies, lack of funding for conducting regular health examination surveys, omission of monitoring practices and capacity scarcity are among the greatest challenges faced by small countries to effectively measure health outcomes. Nevertheless, we identified means of how these can be overcome. For example, the creation of public interdisciplinary repositories enables easily accessible data that can be used for health policy and strategic planning. Health policy-makers, funders and practitioners can consider the use of regular health examination surveys and other tools to effectively manage diabetes at the population level.

## Key messages

- Most small member states have national diabetes plans, national registers and strategies.
- Diabetes plans exist in Cyprus, Malta and Montenegro, countries that have relatively higher diabetes prevalence rates and burden of disease compared to the much lower diabetes burdens in Luxembourg and Iceland.
- Regular health examination surveys are currently missing and, as part of monitoring and evaluation, this is something that should be considered.

## Introduction

Noncommunicable diseases (NCDs) are global epidemics that contribute to a substantial burden related to morbidity, disability and premature death. Diabetes falls within the top five predominating NCDs across the world, including Europe [1]. In 2019, the International Diabetes Federation (IDF) reported an estimated 463 million adults (aged 20–79 years) suffering from diabetes worldwide, with 59 million residing in Europe [2]. By 2045, a 15% rise in the diabetes population is expected to occur within Europe [2].

With the global spread of the coronavirus disease 2019 (Covid-19) pandemic in 2020, several mitigation legislations have been implemented by almost all countries across the globe to safeguard the health and safety of their populations. A number of these legislations have an effect on the healthcare systems and previously established services, including diabetes screening, prevention and management [3]. Although it is too early to assess the exact impact of this pandemic on the diabetes situation across Europe, it can be predicted that the relationship between diabetes and the healthcare services can only be negative in the near future, especially in light of the observed relationship between diabetes and Covid-19 [3].

Small countries are known to face distinct challenges related to their health system governance and their health service delivery due to lack of capacity, limited resources, small market size and their constrained ability to implement effective health system reforms [4]. However, this is not always the case. Indeed, recently in the face of the Covid-19 pandemic, the small country of Malta proved to be a prototype for small and large European countries alike in the response to the first wave of the pandemic through health system preparedness and timely measures [5].

Recently, a common path was agreed among the 11 Member States in the Small Countries Initiative of the WHO European Region [6] to foster a collaborative framework, to promote health and reduce health inequities. These goals were based on the alignment of priorities on health policies within the European health policy Health 2020 framework, the development of capacity-building infrastructure to promote health and reduce health inequities, the set-up of supportive and engaging environments for the implementation of the goals and the development of a platform to share learning and experiences.

Considering the extend of the global diabetes epidemic, it was considered paramount to also explore this epidemic from a small-country perspective, while investigating how the associated population-level strategic planning can be improved. The World Bank and the Commonwealth define small countries or states as those with populations under 1.5 million. This definition together with data on the countries participating in the COST Action CA18218–European Burden of Disease Network (burden-eu) [7] allowed us to include Cyprus, Iceland, Luxembourg, Malta and Montenegro in this study. The objective of the study was to perform a comparative assessment of the existing diabetes situation at the population level while assessing the particular country’s governmental-related policy among these five small countries in Europe. The overall aim was to set up an agenda for health policy and strategic action for small countries in dealing with the diabetes epidemic at a country level. More broadly, the goal is to contribute to the overall continuous battle against the diabetes crisis by identifying potential solutions for small countries.

## Methods

### Methodology rationale

The authors are members of the CA18218–European Burden of Disease Network (burden-eu) CA18218 that focusses on mapping the burden of disease at the European level. Our study was based on a simple model of analysing the existing state of diabetes by synthesizing the evidence to make relevant suggestions on disease management and health policy for these countries. The methodology is split into three parts.

The first part is concerned with the context of analysing the five small European countries of Cyprus, Iceland, Luxembourg, Malta and Montenegro. The rationale behind this selection is threefold. Firstly, there is a lack of studies conducted at a national level in these countries and, hence, this provides an appropriate opportunity to study these. Secondly, when compared to other European countries, these small countries have a high prevalence of diabetes and, as such, it was considered appropriate to focus on these countries to shed light on the problem at hand and how it can be tackled. Thirdly, when comparing small states, lessons can be learnt not only to help small countries but also to inform actions and decisions in geographically larger countries, with a more decentralized decision-making system in place, or at a regional level, having the same population size as that of an entire small-country state.

The second part of this work involves the documentation of the health-policy status for each of the five small European countries according to the (1) epidemiology of diabetes and other related metabolic abnormalities, (2) burden of diabetes status and (3) diabetes register and national plans. Our proposed study aims to contribute to the aforementioned effort within Health 2020 by identifying gaps, differences and similarities between these countries towards evidence-based health-policy planning and strategic action.

The third part of the study is focussed on the synthesis of the findings of an evidence-based proposal on diabetes management in the following three dimensions: (1) monitoring and evaluation, which covers health examination surveys and their implementation in the five countries; (2) prevention, to draw on existing practices for public health diagnostic and screening practices; and (3) health system performance, to contextualize the impact of care delivery on the disease burden for each population.

**Fig. 1:**
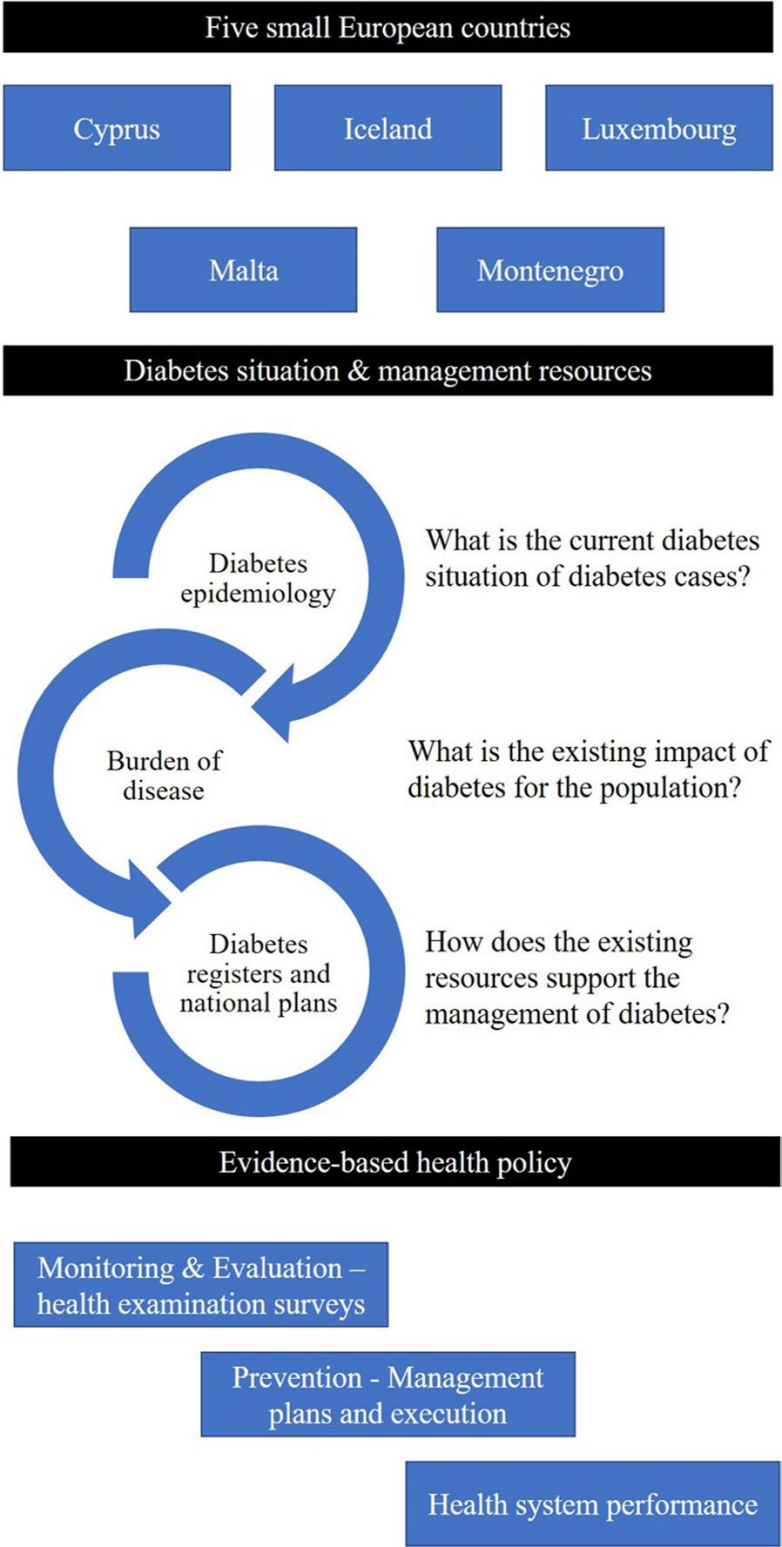
The research methodology

A summary of the methodology protocol can be seen in Fig. 1.

### Data sources

We collected data by contacting Ministry representatives and other governmental bodies in each state, and searched for publicly available information from websites on strategies and policies as available at the respective Ministry of Health, some health sector divisions and appropriate bodies, such as statistical organizations. We identified published articles originating from national surveys and datasets conducted in each of the five countries: Cyprus, Iceland, Luxembourg, Malta and Montenegro. When data were not available online, we contacted the corresponding person who would in turn send us the available nonconfidential data. We searched for documents on national strategies, action plans and/or policies on NCDs and diabetes at a country level.

### Data analysis

The burden of diabetes at a population level was evaluated in terms of the “disability-adjusted life years” (DALYs) metrics as reported by the Institute for Health Metrics and Evaluation (IHME) in their Global Burden of Disease (GBD) study [8]. The same resource was used to evaluate each country’s profile characteristics [8]. Comparisons between the DALYs, years lived with disability (YLDs) and years of life lost (YLLs) data were performed for the five small countries, using the GBD Compare tool [9]. DALYs is an overall measure of the disease burden that takes into account the YLDs and the YLLs due to a disease, which in this case is diabetes. The importance of DALYs is that it provides a consistent, geographic and time-bound metric of population health on both morbidity (YLDs) and mortality (YLLs) measures in units of years. The use of DALYs can assist in relative comparisons between countries for a specific condition to identify gaps in performance, research, health delivery or policy to prevent illness, disability or premature death. It can also be used to guide health system planning and public health interventions within a country for different diseases or by tracking changes for a specific disease over time. Epidemiological data were obtained from the literature in each of the five states for both incidence and prevalence per 100,000 people.

The scientific literature was reviewed by the authors and common frameworks were identified. The data were categorized according to diabetes: prevalence data, diabetes register, governmental strategy and policy and diabetes health care. Subsequently, the published literature, strategies, policies and burden of disease data were reviewed, and an agenda for policy and strategy action based on the small countries experience was formulated.

## Results

Table 1 shows the population characteristics of the five small countries in Europe that were included in the study according to the GBD study for 2017. Although all five countries have comparable life expectancy, intercountry variations are still observed. Cyprus ranked the highest in terms of diabetes mortality at the population level, followed by Montenegro and Malta.

**Table 1:** Distribution of the population characteristics among the five small countries in Europe, 2017 [6]

Population characteristics	Cyprus	Iceland	Luxembourg	Malta	Montenegro
Population	875.9 K	337.5 K	590.5 K	434.5 K	626.3 K
GDP per capital (in $US)	31,531	47,062	97,887	36,920	15,716
Life expectancy at birth (in years)	81.85	82.85	81.65	80.95	76.5
Diabetes death ranking at a population level	5th	> 10th	> 10th	8th	6th

*GDP* Gross domestic product

### Epidemiology of diabetes and other related metabolic abnormalities

Diabetes prevalence rates varied across the five small countries, with the highest rates being reported in Malta and Cyprus followed by Montenegro (Table 2) [10–13]. However, it is worth noting that the data obtained from surveys were conducted in different time frames as well as following different study protocols. For example, Montenegro’s diabetes estimate was based on a combination of data obtained from a national register under the patronage of the Institute of Public Health and the primary healthcare information system. A similar picture is observed for the prevalence of obesity, where the highest obesity rates among the five small countries were reported by Malta (Table 2) [12, 14–17]. Yet, data on impaired glucose regulation (impaired glucose tolerance or impaired fasting glucose) and the metabolic syndrome was available only for Luxembourg and Malta (Table 2) [18–23].

**Table 2:** Comparisons between prevalence of the different metabolic abnormalities across the five small countries in Europe

Prevalence	Cyprus	Iceland	Luxembourg	Malta	Montenegro
Type 2 diabetes	10.4% (2005)	6.72% (2005–2011)	9.8% (2016–2018)	10.4% (2014–2016)	10.2% (2014)
Obesity (BMI > 30 kg/m^2^)	25.5% (2008)	26.6% (2017)	19.2% (2016–2018)	34.1% (2014–2016)	25% (2016)
Impaired glucose tolerance	–	–	25.6% (2013–2015)	–	–
Impaired fasting glucose	–	–	–	24.3% (2014–2016)	–
Metabolic syndrome	–	–	28.0% (2008–2009)	26.3% (2014–2016)	–

*BMI* Body mass index

### Diabetes register and national plans

**Fig. 2:**
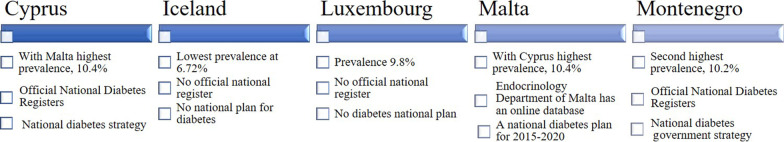
Comparative summary of the diabetes epidemiology and official diabetes registers and plans across the five small countries in Europe

Official national diabetes registers are only present in Cyprus and Montenegro. Malta’s national diabetes register is still in the pipeline. However, the Endocrinology Department of Malta’s state hospital has an online database that physicians can voluntarily input diabetes patients’ data. This database can act as the foundation for the construction of a national diabetes register [24]. National diabetes plans have only been established in Cyprus, Malta and Montenegro [25, 26]. A comparative summary of the official diabetes registers and plans across the five small countries can be observed in Fig. 2.

### Cyprus

In Cyprus, diabetes is high on the health policy agenda due to its impact on the economy and productivity [25]. A national diabetes strategy (2016–2024) is in place and it is based on five pillars: (1) prevention, (2) early detection and care, (3) rehabilitation, (4) diabetes registry and (5) research. An interdisciplinary body, the National Diabetes Committee, has been appointed by the Ministerial Board in order to execute the policy of this diabetes strategic plan. There are 12 institutions carrying out research on diabetes on the island; the University of Nicosia, Nicosia General Hospital and the University of Cyprus are among the top three. Although the island has are no clinical practice guidelines, the specialists follow European guidance or that of the UK. Cyprus has a high diabetes prevalence, but the amount of research done in the field is not appropriate to the needs of the population [27].

In Cyprus, individuals suffering from diabetes are entitled to a holistic healthcare service that includes free medication and consultations.

### Iceland

In Iceland, the position of diabetes within the political agenda is a delicate one. Existing national policies target diabetes-related diseases and risk factors, including obesity, overweight, physical inactivity, smoking and unhealthy eating, but not prevention of diabetes. In fact, there is no national plan for diabetes [25]. However, individuals diagnosed with diabetes are eligible for free medication with a minimal fee for check-up visits to diabetologists, diabetes nurses, ophthalmologists and podiatrists. The diabetes care in Iceland follows the American Diabetes Association guidelines [28]. An interdisciplinary working group appointed by the Minister of Health has recently suggested a national diabetes register for Iceland [29].

### Luxembourg

In Luxembourg there is no diabetes-specific national plan but rather a general national prevention policy that targets the different chronic NCD risk factors, including diabetes, obesity and overweight, dietary habits, smoking, physical inactivity and the harmful use of alcohol [25, 30]. The “Eat Healthy and Move More 2018–2025 Action Plan” is an interministerial prevention strategy developed by the Education, Family, Sports and Health Ministries, with the aim of promoting a healthy diet and physical activity. Diabetic patients are eligible for free medication. However, there are no systematic yearly check-ups organized with endocrinologists or other allied health professionals. The patients need to organize follow-up consultations on their own initiative. Those opting to have check-up consultations will get a percentage reimbursement by the government for the incurred fee. In addition, a national plan for 2019–2023 to fight cardioneurovascular diseases is being implemented, with a specific focus on diabetes risk.

### Malta

In Malta, a national diabetes plan was set up entitled “Diabetes: A National Public Health Priority: 2015–2020” [31]. This plan complemented the already existing “Diabetes Shared Care Programme” [32]. This programme follows a multidisciplinary team effort to provide a free holistic care plan to the diabetes population and includes: regular follow-ups with a diabetes nurse, diabetologist, general practitioner with a special interest in diabetes, dietician, ophthalmologist, ophthalmic nurse and podiatrist. Furthermore, all individuals diagnosed with diabetes are entitled to free medication as well as to a limited amount of blood glucose monitoring strips every month. Additionally, there are a number of preventive strategies and action plans that target the different risk factors for diabetes including the “Healthy Weight for Life Strategy”, the “Noncommunicable Disease Control Strategy for Malta” and the “Food and Nutrition Policy and Action Plan for Malta”.

### Montenegro

In Montenegro, a governmental “Strategy on Healthcare of People who Live with Diabetes 2016–2020” with Action Plan 2017–2020 is under completion [26]. The main objectives of this strategy are to improve the health of these people through effective measures, including early detection, control, treatment and prevention of associated complications. A multisectoral approach is in place to safeguard the adherence and maintenance of this strategy. A national strategy for the prevention and control of NCDs (2008–2020) is also set in place, including the “Master Plan for Health System Development (2015–2020)” focussing, among other things, on priorities for diabetes. Montenegro has other strategies, including the “National Strategy for Sustainable Development—NSSD 2016–2030” and the “Strategy for Healthcare Quality Improvement and Patient Safety 2019–2023”, with both strategies targeting NCDs. Individuals with diabetes are eligible for free medication and healthcare services under the Compulsory Health Insurance Act.

### Burden of diabetes

On assessing the DALYs metric for the diabetes situation across the five small countries in Europe, we draw on differences and similarities, as shown in Table 3. Montenegro had the highest diabetes DALYs and YLDs for 2017 compared to the other small countries; however, Cyprus had the highest YLLs for the same year.

**Table 3:** Distribution of the burden of disease metrics for diabetes across the five small countries in Europe for 2017 [7]

GBD burden of disease study (2017)	Cyprus	Iceland	Luxembourg	Malta	Montenegro
DALYs per 100,000	1098	526.98	786.65	1172.67	1254.47
YLDs per 100,000	600.66	429.75	639.76	776.97	847.71
YLLs per 100,000	497.34	97.23	146.89	395.7	406.76

*DALYs* Disability-adjusted life years, *GBD* GlobalBburden of Disease, *YLDs* years lived with disability, *YLLs* years of life lost

## Discussion

Diabetes is a growing epidemic that gives rise to several different challenges and imposes a substantial burden on healthcare services. This has led to a number of collaborative reports and joint actions between countries and states that are members of the EU with the aim of addressing this epidemic [33]. Across Europe, many countries have introduced national diabetes plans or NCD strategies as part of a national effort to contain this epidemic. We identified a wealth of information related to the strategies and action plans that are related to (1) monitoring and evaluation aspects, (2) prevention and (3) health system performance.

A multistakeholder approach is key when developing such strategies, in addition to a sustainable political leadership [1, 34]. However, political support for a specific framework allocated only to diabetes is not always in place, as seen Iceland, which has the highest gross domestic product (GDP) of the five small states studied. On the other hand, national diabetes plans are already established in Cyprus, Malta and Montenegro. These latter three small countries have relatively higher diabetes prevalence rates and burden of disease compared to a much lower diabetes burden in Luxembourg and Iceland. Nevertheless, all five small countries have an established preventive strategy targeting the various diabetes risk factors and NCDs.

### Monitoring and evaluation

The key to successful NCD strategies is the continuous monitoring and evaluation of the situation at a population level [1]. This is maintained by undertaking regular health surveys, ideally through health examination surveys. Without regular updated evidence-based data, there will be sparse evidence on whether the strategies are working efficiently and whether there is a need for more rigorous interventions. Conducting regular health examination surveys is one of the many challenges faced by most small countries due to the lack of human resources and research budget allocation. In fact, over the past 15 years, a national representative health examination survey covering diabetes was conducted only once in Cyprus and Malta, never in Iceland and Montenegro, while in Luxembourg health examination surveys were performed on a regular basis (2007–2008, 2013–2015 and 2016–2017) [13, 35–37]. The European Health Interview Survey consists of a number of health modules, including a self-reported medical history of diabetes, and is conducted every 5 years among the EU countries, including Iceland [38]. This type of survey depends on self-reporting; hence, it is prone to incorrect recall information biases. However, it still constitutes a good source of information on health indicators at the population level, with reasonable resources.

### Prevention: management plans and execution

An integrated approach is required when targeting chronic diseases, including diabetes [39]. Prevention is one of the key priorities in terms of reducing the burden of diabetes within the population [1]. In fact, prevention is one of the key pillars of the national diabetes plans set up by Cyprus, Malta and Montenegro [31]. Regardless of this focus on prevention, it is of utmost importance that such strategies are adhered to and maintained. Establishing multidisciplinary diabetes care protocols in each country may be the way forward to ensure adherence to such strategies. Nonetheless, it is paramount that adequate human resources, infrastructure and financial budget allocation are present to enhance the healthcare services. In Montenegro, a multisectoral approach has been established to ensure that the strategies are adhered to. However, the implementation of these strategies still presents a major challenge. The small island of Malta together with Cyprus have the highest DALYs burden of the other small countries; they both have one of the highest prevalence rates for both diabetes and obesity [10, 14]. Although Malta has had a National Diabetes Plan in place since 2014, an official diabetes screening framework is not yet present even if various recommendations have been suggested [10, 31, 40]. For Cyprus, the burden of disease data show that YLDs are very similar, about 1.5-fold higher, to data on mortality from diabetes, which possible illustrates that a better diagnosis and regular blood glucose management of the disease can prevent premature deaths. For example, in Malta, it is at the discretion of the family physician or of the individual to seek out regular blood glucose testing and screening for diabetes. A similar picture is seen for Luxembourg.

### Health system performance

A slight discrepancy is observed between the diabetes holistic management approach offered by the healthcare systems of these five small countries. The diabetes populations residing in Cyprus, Malta and Montenegro benefit from a free medication scheme as well as systematic check-up routines. This holistic approach is justified considering the high prevalence of diabetes among each country’s adult population. Regular follow-up enables early detection of complications and a reduction in the healthcare burden. Similarly, the diabetes population in Iceland has the same opportunities, but individuals incur a minimal fee for the check-up consultations. As Iceland has the highest GDP per capita of the five small countries studied here, this health regulation may be justified, especially as Iceland has not only the lowest overall disease burden but also the lowest burden from premature deaths attributed to diabetes, compared to the other small states studied. Of all the small countries in this study, Iceland enjoys the lowest overall diabetes’ burden even though it was noted that it lacks regular evidence-based data to allow adequate monitoring of population health. This is in contrast to Luxembourg, which benefits from access to such data but still has a higher diabetes burden among its population. Therefore, the plan for a more organized diabetes management strategy in combination with better diagnostic and surveillance databases can complement the monitoring and control within the provided framework of service delivery to those small countries who suffer the most from diabetes.

### Implications and recommendations

Small states, irrelevant of their GDP (except for Luxembourg) and their health status, appear to have low priorities for regular and ongoing surveys and population research. The potential reasons for this are multifactorial and include: the lack of human resources, minimal allocation of grants and funding for research, different governance priorities, among others. These issues  hinder the mapping of the burden of diabetes and the associated evidence-based policy and strategic action plans. Conducting regular health examination surveys with a low budget and minimal human resources can be achieved within small countries. Malta’s Health Examination Survey conducted by the University of Malta suggests that an epidemiological health examination survey targeting diabetes can be successfully conducted with minimal resources [36]. A “toolkit for the development and implementation of epidemiological surveys in small populations” has been compiled by the University of Malta in collaboration with the World Health Organization for Health Systems and Policies in Small States [41] to illustrate how small countries can use their minimal resources to conduct epidemiological population-based research. A public interdisciplinary repository may also be created where researchers can share their research and data. It is not uncommon that scholars conduct valuable studies as part of an academic endeavour, but the outcomes are not publicly shared with stakeholders. In small countries, such studies are more likely to be population-based and, if easily accessed, the data can be used effectively for health policy and strategic planning. However, the mechanisms for the translation of this evidence into action are unclear. The collection of accurate health-related data from healthcare facilities can be more feasible in small countries than in larger ones due to the small population size. On the other hand, small countries lack the capacity to conduct local burden of diseases studies. Therefore, the estimated DALYs obtained from the GBD study is a useful metric that helps policy-makers to identify the impact of a disease, such as diabetes, on their country. Additionally, policy-makers can make use of such administrative data collection systems when implementing plans to assist with their decisions. It is, therefore, important that such resource metrics are brought to the attention of policy-makers while setting agendas for health policies and strategic action plans.

Another key challenge that small countries face is the inability to monitor and evaluate community diabetes public awareness while building up the capacity to measure the health outcomes following interventions [33]. Provided that the right tools and data are established, small countries are in a better position to implement interventions and monitor their outcomes due to their small population size. Awareness of diabetes and its associated risk factors is not an adequate stand-alone strategy; rather, it needs to be incorporated into multisectorial interventions that also consider the environment, social status and cultural setting of the population [42]. Therefore, it is of immense importance that policy-makers work with various stakeholders, including local researchers, to collectively map the country-specific burden of diabetes and set the agenda for population-specific action plans.

### Study limitations

The focus was on diabetes management among small countries as associated with health policy and strategic action. This article is based on research literature freely available in online databases as well as on Ministry of Health and other releavent websites and data provided from the appropriate bodies in each of the countries. Ongoing or unpublished studies have, therefore, not been included. As such, the value of this work is limited in these countries (*n* = 5) and on their characteristics of setting health policies and implementing management practices on diabetes. This limitation may prevent the translation of the relevance or value of the current findings on strategic planning and health policy on health promotion and disease prevention for diabetes to the remainder of the excluded countries (*n* = 6) from the European countries of the WHO Small Countries Initiative (*n* = 11). Additionally, further research on addressing these differences could, therefore, bring more clarity on the importance of the context, with emphasis on factors and performance indicators of small countries in the European region.

We acknowledge that the article is based on the five small countries in Europe and that the generalizability of the findings to other smaller European or other countries may not be appropriate. Further research is merited to examine the transfer of knowledge across different small countries. Although the article is based solely on these five small European countries, we cannot draw any conclusions on differences in the country healthcare system and structure. A potentially useful model in future studies might be the Pandemic Risk Exposure Measurement (PREM) to identify factors that are associated with demographic characteristics, measures of a country’s activities and economic and social susceptibilities [43]. For example, we can identify such complex variables to map the impact of diabetes management against the response, policies and strategies that a country can apply during a pandemic situation, such as Covid-19.

### Study strengths

To our knowledge, this is the first study that has focussed on mapping the health policy and strategy on diabetes management in five small countries in Europe. The importance of this work is that it lays the foundation for a future comparative study between smaller and medium-sized or larger European states, and for setting an agenda relevant to them. By collecting and analysing epidemiological, burden of disease and strategy plans we have provided a comprehensive analysis of the health policy landscape related to diabetes. Our results point out the similarities and differences between these countries and subsequently set up an evidence-based agenda for a health-policy framework. Additionally, this study brings together evidence from multiple national sources, highlighting gaps in the process of health policy and strategic action. These can, in turn, be used by policy-makers in each of the countries to inform their existing processes and current practices on diabetes management.

## Conclusions

The diabetes epidemic affects every nation including small countries in Europe. We identified policy documents, strategies and action plans as well as mapped the diabetes situation and the resources for Cyprus, Iceland, Luxembourg, Malta and Montenegro. Despite their small geographical size, we observed distinct challenges faced by these small countries as related to their diabetes epidemiology, burden of disease and diabetes registers and national plans. Iceland and Luxembourg have the lowest prevalence among the five member states, and the lowest burden from premature deaths, despite being the only two member states that have no official national register and no national plan for diabetes. While Malta, Montenegro and Cyprus provide a free holistic care plan to patients diagnosed with diabetes, they also have the highest disease burden from premature mortality. These findings may also (a) justify the need to perform health examination surveys for better monitoring and evaluation, (b) acknowledge gaps in the prevention aspects and set-up of appropriate health population priorities and (c) identify lack of coherent management approaches within the health system to support the population they serve. The key to mapping the burden of diabetes depends on up-to-date evidence-based data, appropriate infrastructure and healthcare frameworks supported by the governance and multisectoral stakeholders. Such an agenda can enable the implementation of targeted health policies and strategic action plans to reduce the burden of diabetes at the population level.

## Data Availability

All data used are provided within the manuscript.

## Abbreviations

DALYs: Disability-adjusted life years
GBD: Global Burden of Disease
IDF: International Diabetes Federation
IHME: Institute for Health Metrics and Evaluation
NCDs: Noncommunicable diseases
WHO: World Health Organization
YLDs: Years lived with disability
YLLs: Years of life lost

## References

[1] Beaglehole R, Bonita R, Horton R, Adams C, Alleyne G, Asaria P (2011). Priority actions for the non-communicable disease crisis. Lancet (London, England).

[2] International Diabetes Federation. IDF diabetes atlas. 9th ed. Brussels, Belgium; 2019. http://www.diabetesatlas.org.

[3] Cuschieri S, Grech S. COVID-19 and diabetes: the why, the what and the how. J Diabetes Complicat [Internet]. 2020;107637. http://www.ncbi.nlm.nih.gov/pubmed/32456846. 10.1016/j.jdiacomp.2020.107637 PMC7242955 32456846

[4] Azzopardi-Muscat N, Funk T, Buttigieg SC, Grech KE, Brand H. Policy challenges and reforms in small EU member state health systems: a narrative literature review. Eur J Public Health. 2016. p. 916–22. https://pubmed.ncbi.nlm.nih.gov/27335326/. 10.1093/eurpub/ckw091 27335326

[5] Cuschieri S (2020). COVID-19 panic, solidarity and equity—the Malta exemplary experience. J Public Health (Bangkok).

[6] Iceland M, Cyprus M, Luxembourg M, Marino S. Implementing the Health 2020 vision in countries with small populations: The San Marino Manifesto. https://www.euro.who.int/__data/assets/pdf_file/0017/310409/San-Marino-Manifesto-en.pdf.

[7] Devleesschauwer B (2020). European burden of disease network: strengthening the collaboration. Eur J Public Health.

[8] Institute for Health Metrics and Evaluation. Global burden of disease (GBD) study. 2020. http://www.healthdata.org/.

[9] Institute for Health Metrics and Evaluation (IHME). GBD Compare | IHME Viz Hub. 2017. https://vizhub.healthdata.org/gbd-compare/.

[10] Cuschieri S (2020). The diabetes epidemic in Malta. S East Eur J Public Health..

[11] Thorsson B, Eiriksdottir G, Sigurdsson S, Gudmundsson EF, Bots ML, Aspelund T (2018). Population distribution of traditional and the emerging cardiovascular risk factors carotid plaque and IMT: the REFINE-Reykjavik study with comparison with the Tromsø study. BMJ Open Br Med J.

[12] Alkerwi A, Pastore J, Sauvageot N, Le Coroller G, Bocquet V, d’Incau M (2019). Challenges and benefits of integrating diverse sampling strategies in the observation of cardiovascular risk factors (ORISCAV-LUX 2) study. BMC Med Res Methodol.

[13] Loizou T, Pouloukas S, Tountas C, Thanopoulou A, Karamanos V (2006). An epidemiologic study on the prevalence of diabetes, glucose intolerance, and metabolic syndrome in the adult population of the Republic of Cyprus. Diabetes Care.

[14] Cuschieri S, Vassallo J, Calleja N, Camilleri R, Borg A, Bonnici G, et al. Prevalence of obesity in Malta. Obes Sci Pract. 2016. p. 466–70. http://www.ncbi.nlm.nih.gov/pubmed/28090352. 10.1002/osp4.77 PMC5192534 28090352

[15] Landlæknir. The Directorate of Health Iceland. 2014. https://www.landlaeknir.is/english/.

[16] World Health Organization Regional Office for Europe. Nutrition, physical activity and obesity Cyprus demographic data prevalence of overweight and obesity (%) among Cypriot adults based on WHO 2008 estimates. 2013. http://www.euro.who.int/en/nutrition-country-profiles.

[17] World Health Organization. Montenegro. 2016. https://www.who.int/nmh/countries/mne_en.pdf?ua=1.

[18] Bocquet V, Ruiz-Castell M, de Beaufort C, Barré J, de Rekeneire N, Michel G (2019). Public health burden of pre-diabetes and diabetes in Luxembourg: finding from the 2013–2015 European Health Examination Survey. BMJ Open.

[19] Cuschieri S, Grech S (2020). Assessing impaired fasting blood glucose criteria for high-risk dysglycaemic populations: an experience from a European population state. J Diabetes Metab Disord.

[20] Cuschieri S, Vassallo J, Calleja N, Pace N, Mamo J (2017). The effect of age, gender, TG/HDL-C ratio and behavioral lifestyles on the metabolic syndrome in the high risk Mediterranean Island population of Malta. Diabetes Metab Syndr Clin Res Rev.

[21] Alkerwi A, Donneau A-F, Sauvageot N, Lair M-L, Scheen A, Albert A (2011). Prevalence of the metabolic syndrome in Luxembourg according to the Joint Interim Statement definition estimated from the ORISCAV-LUX study. BMC Public Health.

[22] Martinovic M, Belojevic G, Evans GW, Lausevic D, Asanin B, Samardzic M (2015). Prevalence of and contributing factors for overweight and obesity among Montenegrin schoolchildren. Eur J Public Health.

[23] Cojić MM, Cvejanov-Kezunović L, Stanković J, Kavarić N, Koraćević M, Damjanović L. Control of glycemia and cardiovascular risk factors in patients with type 2 diabetes in primary care in Montenegro. Facta Univ Ser Med Biol. 2018;0:016. http://casopisi.junis.ni.ac.rs/index.php/FUMedBiol/article/view/3638.

[24] Azzopardi J, Fava S. The Department of Health Diabetes Mellitus Health Information Technology Database in Malta. Malta Med Sch Gaz. 2019;3:40–5. https://www.mmsjournals.org/index.php/MDHG/article/view/181.

[25] Felton A-M, Hall M (2015). Diabetes in Europe policy puzzle: the state we are in. Int Diabetes Nurs.

[26] Ministry of Health Montenegro. Strategies. 2020. http://www.mzdravlja.gov.me/en/library/strategije?alphabet=lat.

[27] Pallari E, Lewison G, Pallari CT, Samoutis G, Begum M, Sullivan R (2018). The contribution of Cyprus to non-communicable diseases and biomedical research from 2002 to 2013: implications for evidence-based health policy. Heal Res Policy Syst.

[28] American Diabetes Association AD (2020). Glycemic targets: standards of medical care in diabetes-2020. Diabetes Care.

[29] Ministry for Health Iceland. Suggestions for responding to the growing incidence of diabetes. 2018. https://www.stjornarradid.is/efst-a-baugi/frettir/stok-frett/2018/04/20/Tillogur-um-vidbrogd-vid-vaxandi-nygengi-sykursyki/.

[30] Sante Luxembourg. GIMB ¦ Promotion de l’alimentation saine et de l’activité physique 2018–2025—Portail Santé//Grand-Duché de Luxembourg. 2006. https://sante.public.lu/fr/politique-sante/plans-action/gimb-2018/index.html.

[31] Parliamentary Working Group on Diabetes. Diabetes: A National Public Health Priority. Proposal for a National Strategy for Diabetes 2015–2020. Malta; 2015.

[32] Ministry for Health Malta. Protocol for Shared Care Diabetes in Malta. https://deputyprimeminister.gov.mt/en/phc/Pages/Services/Diabetes-Shared-Care-Programme/Protocol.aspx.

[33] Richardson E, Zaletel J, Nolte E. POLICY BRIEF National diabetes plans in Europe. What lessons are there for the prevention and control of chronic diseases in Europe? On behalf of Joint Action CHRODIS HEALTH SYSTEMS AND POLICY ANALYSIS. 2020. www.nijz.si.

[34] Cuschieri S (2019). Type 2 diabetes—an unresolved disease across centuries contributing to a public health emergency. Diabetes Metab Syndr Clin Res Rev.

[35] Finnish Institute for Health and Welfare. European Health Examination Survey. 2019. http://www.ehes.info/national/national_hes_status.htm.

[36] Cuschieri S, Vassallo J, Calleja N, Pace N, Mamo J. Diabetes, pre-diabetes and their risk factors in Malta: a study profile of national cross-sectional prevalence study. Glob Health Epidemiol Genomics. 2016;1. 10.1017/gheg.2016.18 PMC5870414 29868212

[37] Alkerwi A, Sauvageot N, Donneau A-F, Lair M-L, Couffignal S, Beissel J (2010). First nationwide survey on cardiovascular risk factors in Grand-Duchy of Luxembourg (ORISCAV-LUX). BMC Public Health.

[38] EUROSTAT. European Health Interview Survey—Eurostat. 2020. https://ec.europa.eu/eurostat/web/microdata/european-health-interview-survey.

[39] European Diabetes Leadership Forum. The diabetes epidemic and its impact on Europe. Copenhagen; 2012. https://www.oecd.org/els/health-systems/50080632.pdf.

[40] Cuschieri S, Vassallo J, Calleja N, Pace N, Abela J, Ali BA (2016). The diabesity health economic crisis-the size of the crisis in a European island state following a cross-sectional study. Arch Public Health.

[41] Cuschieri S, Calleja N, Mamo J. Toolkit for the development and implementation of epidemiological surveys in small populations report. WHO collborative centre, University of Malta Islands & Small states Institute, Msida, Malta. 2019.

[42] Cuschieri S, Grech S (2019). Closing the gap—is type 2 diabetes awareness enough to prevent the growing epidemic?. Diabetes Metab Syndr.

[43] Grima S, Kizilkaya M, Rupeika-Apoga R, Romānova I, Dalli Gonzi R, Jakovljevic M (2020). A country pandemic risk exposure measurement model. Risk Manag Healthc Policy.

